# The Relationship of Pre and Early Postnatal Risk Factors with Breast Cancer

**DOI:** 10.31557/APJCP.2020.21.1.75

**Published:** 2020

**Authors:** Atieh Akbari, Maryam Khayamzadeh, Mohammad Esmail Akbari, Mohammad Reza Sohrabi, Ladan Ajori

**Affiliations:** 1 *Cancer Research Center, *; 2 *Social Determinants of Health Research Center,*; 3 *Department of Obstetrics and Gynecology, Shohada Tajrish Hospital, Shahid Beheshti University of Medical Sciences, Tehran, Iran. *

**Keywords:** Having been breastfeed, family history, breast cancer, Iran

## Abstract

**Background:**

Breast cancer (BC) is the most prevalent cancer in Iranian women and the fifth most common cause of cancer-related death in Iran. Risk factors in the adult life may act during fetus life and after delivery. We conducted a case–control study to find out the relation of in utero and early life exposure and risk of BC.

**Methods:**

A structured questionnaire that covered demographic criteria and BC risk factors in utero was completed for case (732 cases) and control (584 subjects) groups, matched in terms of demographic variants, reproductive issues and socioeconomic status. Odds ratio (OR) and 95% confidence intervals (CI) were computed as measures of association from the logistic models.

**Results:**

Having been breast feed for more than 19-24 month (P<0.001, OR 0.03, CI 0.004-0.21) is protective and positive family history of mother (P-value= 0.009, OR 3.4) is a risk factor for BC in adult.

**Conclusion:**

There is increasing recognition that condition in utero is important for later risks in breast. Emerging evidence suggests an association between intrauterine status and women prenatal condition and their subsequent risk of developing breast cancer. this is the first Iranian study assessing prenatal factors and breast cancer risk in the EMR and it should be followed by the larger group of cases and controls in the future.

## Introduction

Many individual risk factors affect patients with breast cancer. Diet, physical activity, alcohol consumption, smoking, endogenous sex hormone variation and exogenous using of estradiol hormones and its derivate such as diethylstilbestrol (DES), reproductive factors including nuliparity, breastfeeding, and abortion are some preventable risk factors for breast cancer (Akbari et al., 2011). Family history, hereditary gene amplification, and specific gene profile manipulation are other risk factors, which are not preventable in individual cases yet. Also, nowadays, the spirituality and psychosocial determinants are considered more by researchers. 

These risk factors are well known and confirmed by researchers in various studies. The authors published a scientific article, discussing such kinds of factors in Iranian women supported by others in this country (Akbari et al., 2011). Breast cancer is the most prevalent cancer among Iranian women with the Age Specific Rate (ASR) of 30/100,000 normal population worldwide with different incidence. It is the fifth cause of death due to malignancy. (Ministry of Health and Medical Education, 2007).

Although, there are some prenatal factors as breast cancer risk factors, limited articles discussed it throughout the world and to the best of our knowledge, no study has discussed prenatal and early postnatal risk factors related to breast cancer in the Eastern Mediterranean Region (EMR). The important prenatal and early postnatal factors are as follow: 

Gestational age, mother age at first pregnancy, birth weight, smoking, alcohol consumption by mother during pregnancy, birth order, twin status, eclampsia and pre-eclampsia, family history of mother and some genetic and epigenetic event, maternal age, being breastfeed ‘which is considered as an early postnatal protective risk factor’ and its duration (Trichopoulos et al., 1990; Weidman et al., 2007).

Investigating such factors after decades may be difficult and prohibiting factor for researchers. The present study is the first case control study, assessing prenatal and early postnatal factors and risk of breast cancer in the EMR.

## Materials and Methods

The present cross sectional case control study was conducted during January 2014 to April 2017 in the Cancer Research Center, Shahid Beheshti University of Medical Sciences (CRC, SBUMS), Tehran, Iran.

A total of 1 316 individuals participated in this study, 732 of whom suffered from breast cancer and 584 of whom were healthy individuals. They were consisted of women aged between 25 to 55 years old; their diseases were confirmed pathologically, and they were pursuing their treatment process at CRC SBUMS during January 2014 to April 2017. They were asked about being breastfeed during infancy, family history of BC in mother, maternal ages at first pregnancy, gestational age, birth weight, twin status, mother smoking during pregnancy, type of delivery, mother’s job, family income at that time, psychological disorder and having bad event during mother life or pregnancy time. Early postnatal was defined as the first 2 years after delivery.

No participant in control group was involved with any kind of malignancy, especially breast cancer; their health was confirmed by investigating clinically, imaging (Mammogram, Sonogram, MRI if needed), and even by pathological assessment. The study was approved by ethical committee and individual cases. (No: IR.SBMU.MSP, REC, 1395.133). A well-established questionnaire made by the authors and filled up by educated staff was used in the study as the research tool after validation during 3 years’ study. The data were analyzed by SPSS version 20 with CI 95% and P- Value <0.05.

**Table 1:** Having been Breastfeed During Infancy in Case and Control Gro

Having been Breastfeed	Benign (%)	Malignant (%)	Total (%)	P-value	OR	Lower CI	Upper CI
<6 month	68 (53.1)	60 (46.9)	128 (100)	0.1			
7-12 month	48 (4602)	56 (53.8)	104 (100)	0.07	0.26	0.06	1.14
13-18 month	80 (90.9)	8 (9.1)	88 (100)	0.17	0.35	0.07	1.57
19-24 month	388 (53.6)	336 (46.4)	724 (100)	0.001	0.03	0.004	0.21
>24 month	12 (23.1)	40 (76.9)	52 (100)	0.04	0.26	0.06	0.97
Total	596 (54.4)	500 (45.6)	1096 (100)				

**Table 2:** Logistic Regression Analysis of Prenatal Risk Factors Influencing Breast Cancer

Having been Breastfeed	P-value	OR	Lower CI	Upper CI
<6 month	0.1			
7-12 month	0.95	0.28	0.06	1.24
13-18 month	0.16	0.34	0.07	1.56
19-24 month	0.001	0.02	0.004	0.18
>24 month	0.04	0.25	0.06	0.95
Family history	0.01	4.16	1.3	13.27

## Results

As shown in Table 1, being breastfeed during infancy reduced the risk of breast cancer significantly if the duration per infant was 18 to 24 months or more. Family history of mother regarding breast cancer was significantly affecting the cases with OR = 3.4 and P = 0.009, compared to benign individuals. Maternal ages at first pregnancy and during pregnancy were not significant between patients with breast cancer (P = 0.4) and healthy individuals (P = 0.8).

Birth weight, which was defined as less than 2,500 g, 2,500 to 4,000 g, and more than 4,000 g, were not significant as the risk factor for breast cancer (P = 0.3). Gestational age for the case and control groups, assessed as pre-term labor (more than 4 weeks earlier than the estimated time), term, and post-term labor (more than 4 weeks after the estimated time), was significantly different (P = 0.003) .

Twin status, mother smoking during pregnancy, type of delivery (Vaginal-C/S), mother job, family income at that time, psychological disorder, and having bad event during mother life or pregnancy time were not significant (P = 0.4, 0.26, 0.32, 0.29, 0.29, 0.83, and 0.82, respectively). In the multivariate analysis, being breastfeed during infancy with duration of 24 months or more per child and/or 19 to 24 months was significant. Also, the family history was significantly different between cases and controls. (Table 2).

## Discussion

Some limited studies assessed prenatal factors affecting breast cancer incidence worldwide and no study was conducted in the EMR. There is limited discussion in the publication analyzing a hypothesis asking does breast cancer originate in utero? (Trichopoulos et al., 1990).

Some risk factors such as ionizing radiation and diethylstilbestrol (DES) in human beings and chemicals in animals will act as risk factors during mother pregnancy and/or increased concentration of estrogen in pregnancy increases the probability of future occurrence of breast cancer in daughters (Trichopoulos et al., 1990; Weidman et al., 2007; Carpenter and Bedient, 2013).

Cancer originated from both genetic and/or epigenetic changes (Weidman et al., 2007). Disparities in gene expression due to intrauterine life risk factors will affect DNA methylation and chromatin structures responding to the environmental risk factors during life years (Weidman et al., 2007). The epigenetic effect inducing chronic non-communicable diseases such as cancer is not clearly identified and evidently documented, but theoretically it is accepted and well defined (Weidman et al., 2007).

As a consequence, exposures during gestation have been demonstrated to lead to cancer later in life (Carpenter and Bedient, 2013). The most vulnerable ages affect the genes and epigenum consequently, are fetal and prenatal periods followed by first year of life, which is defined as breastfeeding period. During the fetus life, cells are replicating rapidly and if DNA is damaged, prenatal defects may result in and lead to cancer later in life (Carpenter and Bedient, 2013). It is more convenient with exposure to endocrines during the fetus life (Birnbaum and Fenton, 2003). Accumulating such risk factors with producing epigenetic changes will be presented during the adult life (Carpenter and Bedient, 2013; Birnbaum and Fenton, 2003).

Exposing to DES during gestational period as a chemical agent is at the highest risk for cancer comparing with those exposed later in life. DES is a synthetic agent that has been used during pregnancy with false assumption that would reduce the spontaneous abortion (Carpenter and Bedient, 2013). These people will be at risk of breast cancer and uterus adenocarcinoma after age of 40 years (Carpenter and Bedient, 2013). This effect is based on genetic mutation and epigenetic changes. In many studies, natural estrogen is associated with high level in breast, vagina, and uterus in human and animals (Birnbaum and Fenton, 2003).

These are not the only risk factors, also the psychospiritual and social factors will affect the fetus as well as his/her mom (Ariana et al., 2018; Akbari et al., 2016). Our study did not prove the effect of this aspect of health, which may be due to the shortage of number of cases.

This hypothesis is based on 4 assumptions: (Trichopoulos et al., 1990)

1. Estrogen is an important component factor in breast carcinomatosis.

2. Factors which increase the risk of cancer when they act postnatally may also increase the risk of cancer when they act in the utero.

3. Estrogen concentration is at least 10 times higher during pregnancy than other periods of adult life.

4. In pregnancy, estrogen concentration and secretion rates vary widely among individualizes due to exogenous factors.

In a cohort study, the relationship between prenatal DES exposure and breast cancer risk were assessed during follow-up; 102 cases of invasive breast cancer included the study, with 76 women exposed at DES orally for spontaneous abortion and 26 unexposed women (overall age adjusted IRR was 1.40) (95% CI) (Palmer et al., 2006).

The length of gestational and maternal exposure to a high fat diet and alcohol increases the pregnancy estrogen levels and risk of breast cancer (Grotmol et al., 2006; Ekbom et al., 1992; Janerich et al., 1994). In this study, we did not find any relation between them.

Birth weight is another issue highlighted in the literatures as a prenatal risk factor for breast cancer. Low birth weight or normal weight was associated with the decreased incidence of breast cancer in pre-menopausal women (Grotmol et al., 2006; Michels et al., 2006; Hodgson et al., 2004; Potischman and Troisi, 1999). In the present study, there was no relationship between birth weight and breast cancer in future.

Being breastfeed is a challenging issue in different studies. Potichman et al., (1999) reported 20% to 35% risk reduction for breast cancer at least in 4 well-established research studies. Also, Evergreen perinatal education confirmed these natural procedures (Ginna, 2013). Being breastfeed ‘which is considered as an early postnatal protective risk factor’ is significantly protective against breast cancer. The duration of breastfeeding and being breastfeed are important issues in many studies and even in some religious sources. (Jones and Baylin, 2002; Holy Quran).

The duration of breastfeeding for anatomic changes in the mother and dietary beneficence for the infant may be at least 18 months and completed at 24 months (Akbari et al., 2011). This finding is a novel criterion, which is not highlighted in some other studies. In this study, having been breastfeed for 19 to 24 month and more than 24 months were significantly associated with the reduction risk of breast cancer in daughter.

Family history of mother before pregnancy in patients with breast cancer was another challenging issue, conducted in Korea. The relationship between family history of mother and risk of breast cancer was significantly positive (Park et al., 2008). Family history of mother regarding breast cancer in our study was significantly prominent in patients with breast cancer (P = 0.009, OR = 3.4). Family history of mother and working during pregnancy as an intrauterine risk factor for fetus seems to be due to some genetic or epigenetic events due to intrauterine life (Ekbom et al., 1992; Jones and Baylin, 2002). However, Janreich et al., (1994) did not find any relationship between family history of mother and working during pregnancy and risk of breast cancer. 

Trichopoulos et al., (1990) reported that blood concentration of estrogen in pregnancy is significantly lower (30%) in young mothers aged less than 20 years than older ones aged 20 to 29 years. This fact was confirmed in more than 53 reviewed articles. (Grotmol et al., 2006; Hodgson et al., 2004; Sanderson et al., 2006). We did not find any relationship between age at pregnancy and risk of breast cancer in daughters in our study; the reason may be due to the few number of moms younger than 20 years old. 

In our study, being breastfeed was significantly different between case and control groups and in particular the duration of feeding between 16 to 24 months and more than 24 months was significant (P = 0.001 and P = 0.04, respectively). The duration data are similar to our previous research on breastfeeding during adulthood. (Akbari et al., 2011). Maternal age was not significant in case and controls (P = 0.8), regarding its efficacy in many other studies.

Gestational age in our study was significant for the pre-term labor as a risk factor for breast cancer (P = 0.003); many translate the non-appropriate cell differentiation during immature pregnancy period. Regarding these events, oncogenes, tumor suppressor genes, and DNA repairs genes are active members causing cancer.

Oncogenes act in a dominant manner, tumor suppressor genes act in a recessive manner and the function of both alleles must be lost to increases the probability of cancer development. This indicates that it takes time to accumulate enough genetic damage and/or epigenetic manipulation to develop the cancer. Generally, these changes are more common in people with positive family history. The individual susceptibility for cancer may be acquired in utero or in early life when the organs are still developing (Grotmol et al., 2006)

In our study maternal age (P = 0.8), birth weight (P = 0.3), and twin status (P = 0.4) were not significant breast cancer risk factors. However, high maternal age at first pregnancy in Sanderson et al., (2006) study showed increase in the germ cell mutation and repair DNA in offspring, so increased the risk of breast cancer in first child. Also, Trichopoulos et al., (1990) and Ekbom et al., (1992) showed higher level of estrogen in >20 year-old women. 

Janerich et al., (1994) and Hodgson et al., (2004) found no correlation between maternal age at first pregnancy and risk of breast cancer in offspring. In a met analysis published in 2008, 6 studies found strong correlation between higher birth weight and more exposure to estrogen and IGF-1that increases the risk of breast cancer (Michels et al., 2006; Potischman and Troisi, 1999; Park et al., 2008), however 2 studies did not find any correlation (Park et al., 2008) and 2 studies had inverse correlation (Park et al., 2008).

In Park et al.,’s (2008) study. and Sanderson et al.,’s (2006) study, twin with higher estrogen in pregnancy increases the risk of breast cancer [5 study had strong relationship between twin and breast cancer, 8 study had a week relationship, and 4 study had a small decrease] (Park et al., 2008). Regardless of many effects in developing specific cancers, smoking was not significant in our study, which may be due to few number of smokers in both group (56 in control group and 46 in case group, P = 0.83).

We also evaluated the effect of some psychosocial events during pregnancy, such as mother job (P = 0.29), psychosocial disorders (P = 0.83), bad event (P = 0.82), and family income (P = 0.29), which were not significant. 

In multivariate analysis, being breastfed 9 to 24 months or more and family history of mother remained significant.

In conclusion, there is increasing recognition that condition in utero is important for later risks in several organs particularly in breast. Emerging evidence suggests an association between intrauterine status and women prenatal condition and their subsequent risk of developing breast cancer. Some of these criteria vary in mothers because of different hormone levels, proliferative indexes, germ cell mutation, formation of cancer stem cells, and other genetic and epigenetic events. Studying breast cancer prevention and management should be started from intrauterine life through life long period.

*Recommendation*

To the best of the researcher’s knowledge, this is the first Iranian study assessing prenatal factors and breast cancer risk in the EMR and it should be followed by the larger group of cases and controls in the future, epidemiologically and biologically including genetic and epigenetic variation during life after exposing to some risk factors during fetus life.
